# Effects of pretreatments of banana (*Musa AAA,*Omini) on the composition, rheological properties, and baking quality of its flour and composite blends with wheat flour

**DOI:** 10.1002/fsn3.378

**Published:** 2016-05-11

**Authors:** Adegoke H. Bakare, Olukemi D. Ogunbowale, Mojisola O. Adegunwa, Joseph O. Olusanya

**Affiliations:** ^1^ Department of Hospitality and Tourism Federal University of AgricultureAbeokutaNigeria; ^2^ Department of Food Science and TechnologyFederal University of AgricultureAbeokutaNigeria; ^3^ Department of Home Economics and Hotel ManagementTai Solarin University of Education NigeriaIjebu‐OdeNigeria

**Keywords:** Baking, banana, bread, pretreaments, production, rheology

## Abstract

Effects of chemical and heat pretreatments on the protein, gluten, and alpha‐amylase activity, pasting (Peak [*P*], Final [*F*] setback [*S*] viscosity, pasting temperature [*PT*] and time [*T*]) and alveogram (Energy [*E*], maximum inflation [*MI*], *P*/*L*, and elasticity index [*EI*]) properties of flour from the pretreated bananas and its composite with wheat flour (WF) were examined. The baking (water absorption [WA] and specific volume [SV]) and sensory properties of bread produced from the flour were also examined. Protein, gluten, and alpha‐amylase activity ranged from 4.75 ± 0.07%, 30.25 ± 0.05%, and 4.00 ± 0.05 min to 13.75 ± 0.06%, 35.64 ± 0.06%, and 39.61 ± 1.18 min with WF:PTBF/95:05, WF:CTBF/00:100, WF:BBF/80:20, WF:100 and WF:CTBF/00:100, WF:PTBF/95:05, WF:100, WF:PTBF/00:100 having lowest and highest values, respectively. *P*,* F*,* S* viscosities, *PT* and *T* ranged from 186.17 ± 0.71, 217.08 ± 1.41, 38.92 ± 5.42 RVU, 84.70 ± 0.28°C, 5.04 ± 0.05 min to 461.0 ± 5.07, 348.5 ± 8.84, 88.83 ± 0.24 RVU, 87.20 ± 0.00°C, 6.24 ± 0.05 min, respectively. *E*,* MI*,* P*/*L*, and *EI* ranged from 141.50 ± 0.71 × 10^−4^J, 15.35 ± 0.07, 0.59 ± 0.83 and 35.85 ± 0.07 to 325.00 ± 1.4 × 10^−4^J, 22.55 ± 0.07, 2.75 ± 0.07, and 70.50 ± 0.71, respectively. WA and SV were 48.12 ± 0.07 to 52.60 ± 0.14 and 2.850 ± 0.07 to 5.635 ± 0.18 with the WF having significantly (*P* < 0.05) higher values than other blends and the most acceptable in terms of appearance and taste.

## Introduction

Consumption of wheat‐based products is not likely to decrease, considering the growth in population and urbanization (Bakare 2008; Malomo et al. 2011) and the exotic lifestyle and food habits adopted by the citizens of most of the non‐wheat‐producing countries (Bakare et al. 2015). Bread is a staple food consumed in different parts of the World. It was described as a fermented confectionery product produced mainly from wheat flour (WF), water, yeast, and salt by a series of process involving mixing, kneading, proofing, shaping, and baking (Dewettinck et al. 2008). Inspite of some of the nutritional disorder attributed to the consumption of bread, it is still desired by many people across various socio‐demographic characteristics.

Several studies have reported the prospects of composite flour technology as a means of reducing the dependence on wheat for the production of bakery products (Mepba et al. 2007; Biljan and Bojana 2008; Manuel et al. 2008; Alex et al. 2008; Ade‐Omowaye et al. 2008; Lin et al. 2009; Adeleke and Odedeji 2010; Malomo et al. 2011, 2013; Bakare et al. 2013, 2014a,b, 2015). Partial replacement of WF with flour from suitable indigenous crops remains the only viable practical option for reducing the consumption of wheat, provided that some of the challenges associated with the utilization of composite technology at industrial scale can be minimized (Bakare et al. 2015). It is also necessary to explore the possibility of using crops that are underutilized and with less competing uses, those highly prone to postharvest losses and those that possess strategic nutritional advantages that can be used to alleviate endemic malnutrtional challenges.

Bananas (*Musa* spp) are highly perishable crop produced in the tropical and subtropical countries of the world where they are mostly grown for either home consumption or for the local markets. It is the world's most popular fruit and one of the world's most important staple foods, after rice, wheat, and maize. About 107 million metric tons of bananas were produced in more than 130 countries in 2011 representing a total trade value of US$9 billion (FAO 2013). Nigeria, with an estimated population of 177.5 million people, gross domestic product per capita of US$3,203.3 (World Bank 2013) is one of the largest producer in Africa recording about 2.78 million tons of banana production (FAOSTAT 2013). Africa exported about 649 000 tons of bananas in 2012 representing 3.9 percent of global export with Côte d'Ivoire and Cameroon being the two topmost exporters (FAO 2014).

Bananas are good sources of carbohydrate, vitamins A, B, and C. It is also rich in phosphorus, potassium, magnesium, selenium, and iron. (Wall 2006). Banana contains a large amount of easily assimilable sugars, making it a good source of quick energy and an important means of recovery from fatigue (Pius 1994; Kyamuchangine et al. 2002).

Major limitations to the utilization of banana is its limited shelf life and its rapid susceptibility to postharvest losses, particularly when fully matured, which necessitate the need for processing to a more stable intermediate or ready‐to‐consume forms. Drying is one of the traditional methods used in the tropics for processing bananas into stable storage forms. Drying brings about a substantial reduction in weight and volume; thereby minimizing packaging, storage, and transportation cost and also enable storability of the product under ambient temperature, especially in developing countries (Senadeera et al. 2005). Dried bananas are easy to handle and can be easily incorporated during food formulation and preparation. Hot‐air drying of food materials also help to control product quality, enhance hygienic handling and reduce product losses (Corzo et al. 2008). In addition, food poisoning and spoilage are minimized, because reduction in moisture content often resulted in food with low water activity which in turn enhances bacteriostatic control of the activities of microbes in food.

Common problems associated with drying of tropical fruits included shrinkage of the food cells, browning, and loss of rehydration ability, wettability, and case hardening. Browning is an important physiological disorder that degrades the qualities of fruits and vegetables. Enzymatic browning is a widespread color reaction occurring in fruits and vegetables, which involves the interaction of oxygen, phenolic compounds and polyphenol oxidases (PPO). Browning is usually initiated by the enzymatic oxidation of monophenols into *ο*‐diphenols and *ο*‐diphenols into quinones, which undergo further nonenzymatic polymerization leading to the formation of pigments. Enzymatic browning negatively affects the quality of fresh‐cut fruit and vegetable (Ioannou and Ghoul 2013). Banana is susceptible to enzymatic browning during processing (Luo and Tao 2003) and storage. Browning has a negative effect on their appearance, sensory properties (taste, odor, and texture) and nutritional value (Jiang 2004; Komthong et al. 2006).

Banana is susceptible to enzymatic browning during processing (Luo Y and Tao Y, 2003) and storage. Browning has a negative effect on the appearance, sensory properties (taste, odour and texture) and nutritional value (Jiang, 2004; Komthong et al., 2006) of banana. Some of the factors that determine the rate of enzymatic browning in banana are concentrations of PPO and other phenolic compounds, pH, temperature and availability of oxygen. Techniques and mechanisms that have been explored to control the enzymatic browning are meant to eliminate one or more of these factors. According to Ioannou and Ghoul (2013) some of these techniques included chemical treatments (antioxidants, acidifying, agents of firmness, or chelating agents) and then physical methods (blanching, freezing, and the modification of product atmosphere). It has been noted that acidification may reduce or inactivate the PPO activity, particularly when pH is below 3 (Grimm et al. 2012). Citric acid may act both as acidifying (Ihl et al. 2003) as well as a chelating agent (Jiang et al. 2004). Heat treatment of up to 60°C has been used to induce heat shock which inhibit browning and also provide protection against pathogens (Loaiza‐Velarde et al. 2003; Qiang and Yaguang, 2007). Blanching is a type of heat treatment given to food primarily to inactivate enzyme systems responsible for sensory and other quality alterations in food. Blanching limits the oxidation and may also enhance the colors of some vegetables. It can, however, alter the consistency of treated product and also generate loss of nutrients, particularly when done in water (Mazzeo et al. 2011) instead of steam which is also suitable for high moisture, enzyme active, and soft texture food like banana.

Protein‐starch interaction catalyzed by enzyme‐induced biochemical processes also affects the quality of baked products, generally, and bread in particular.

In summary, bananas are nutritive, highly perishable with limited shelf life. Nigeria is one of the largest producers of banana in Africa, its susceptibility to postharvest losses can be reduced and its utilization can be enhanced by drying and converting bananas to flour. This may increase its versatility and application for different end use, particularly in bakery products like bread. However, because banana is an enzyme active food, suitable pretreatment prior to drying is a necessity. It is therefore necessary to assess the effects of such pretreatments on the functionality of its flour before it can be recommended for use in bread making. This study, therefore, examined the effect of chemical pretreatments and blanching on the proximate and chemical composition of banana flour, their pasting properties, and the rheological and baking qualities of their composite blends with WF.

## Materials and Methods

### Source of materials

Mature green banana (Musa AAA group, Omini) bunches were purchased from an Agriculture produce market in Abeokuta South Local Government area of Ogun State, Nigeria. WF was obtained from the Nigeria Eagle Flour Mills Ltd, Ibadan, Oyo State while other ingredients such as sugar, iodized salt, yeast, fat, and ascorbic acid were purchased in the retail markets.

### Preparation of flour

Banana was processed as described by Bakare et al. (2015) with some modifications. Mature green but unripe banana were de‐fingered, washed, peeled, manually sliced into cylindrical pieces of 2‐mm thickness and samples were subjected to two types of pretreatments; chemical pretreatments with 5% potassium metabisulphite and citric acid solution for 30 minutes, respectively, as well as steam blanching at 100°C for 2 min after which it was blotted dry with clean tissue paper to remove adhered surface water, making a total of three treatments.

The banana slices were placed on a foil paper placed on drying racks to avoid sticking together; it was then dried in a cross‐flow Gallenkamp oven (Model OV‐160 size 2 BS, Weiss Technik UK Loughborough, Leicestershire, U.K.) at 70°C for 18 h. The dried chips were milled to flour in a Disc mill (Model FFC‐15, Shandong‐Jimo Agricultural Machinery, Qingdao City, Shandong Province, China) at 8800 rpm and sieved through a 250 *μ*m mesh sieve (no.60.W.S. 8570 Tyler Blvd, Mentor, OH 44060).

### Analysis of flour

All analyses were performed in triplicates. Proximate, gluten, and water absorption of WF and blends were analyzed with official methods (AACC 2000) using a Partens Inframatic analyzer (Model 9140, SE‐126 53, Hägersten, Sweden). Gluten content was determined according to AACC International method No. 38–12 by using the Glutomatic (Partens Glutomatic system (Model 2200, Instrumentvägen 31 SE‐126 53 Hägersten, Sweden) to measure the gluten quantity and quality of the flour. Alpha‐amylase activity was determined with the Hagberg falling numbers instrument (Partens model no 1500, Instrumentvägen 31 SE‐126 53, Hägersten Sweden, USA), based on (AACC 2000) approved method. Crude fiber content of flour was determined by trichloroacetic acid method as described by Entwisted et al. (1949).

### Viscosity characteristics of flour

Pasting characteristics of the treated flour were determined according to ICC No 162 method. Rapid visco analyser (RVA) series 4 (RVA; series 4, Newport Scientific P.T.V., Warriewood, Australia) with the aid of thermocline for Windows (version 1.1. Software, 1996) was used for the analysis. The 12‐min profile was used for all the analyses. It consists of idle temp of 50°C for 1 min, then 50–95°C in 3 min 45 sec, held at 95°C for 2 min 30 sec, cooled to 50°C over 3 min 45 sec, finally, held at 50°C for 2 min. Two paddle speeds of 960 rpm for the first 10 sec followed by 150 rpm were employed for the remaining duration of the test cycle. The weight per sample used for each analysis was calculated from the formula:

$$\text{Corrected sample weight for RVA}\left(S\right)=\frac{A\times 100}{100-M}$$

(W)=25+(A−S)

where *A* = weight of flour sample: using the RVA manual as a guide.

*S* = corrected sample weight.

*M* = actual moisture content of the sample.

*W* = volume of water used.

### Alveogram chararcteristics

The Alveograph (Chopin NG France) was used to measure (AACC 2010) characteristics that provided insight into the fermentation tolerance of the dough as may be exhibited during proofing stage of bread making. Characteristics of interest that were measured included the average resistance to expansion indicated by the peak height (mm), extensibility indicated by length (*L*) of the alveogram curve, energy input (Joules) required for the mechanical deformation of the dough (*W*), inflation required for maximum development (*G*) and the elastic resistance (*Ie*) of the measured dough samples.

Flour sample (250 g) of known moisture content was placed into the mixer, sodium chloride solution (2.5%) was added through a buret (i.e., 129.4 mL for flour with 14% moisture) and mixed for 7 min. The dough was forced through the extrusion gate in the form of a thin strip on to a small oiled steel plate. Five extruded dough pieces of designated length were cut off, rolled with an oiled rolling pin to a uniform thinness, cut into a circular disk, transferred to an oiled steel plate and subjected to a brief rest period in a tempered compartment of the alveograph for 15 min. Each circular dough test pieces were removed from the compartment and inserted between two metal plates that held it securely in position. The air valve was opened to supply air pressure to the held dough through an orifice. The electrically driven recording manometer was simultaneously activated to record the air pressure inside the dough bubble against time.

### Bread production

Bread loaves were produced according to AACC (2010) with slight modification. Formulations included: wheat/banana (100:00, 90:05, 90:10, 85:15, and 80:20) composite flour 100 g (14% moisture), 9.0 g sugar, 1.1 g iodized salt, 1.8 g yeast, 1.8 g fat, and 20 ppm of ascorbic acid as dough improver.

The bread samples were produced in the laboratory of Nigerian Eagle Flour Mills Ltd, Ibadan‐Lagos Expressway, Ibadan, Oyo State, Nigeria. The dough was mixed using Chaplain spiral mixer (Model BT10C, Caplain Machines 999 rue du Tuboeuf–BP.99–77170 Brie Comte Robert–France), fermented (proofed) at initial and final fermentation time of 15 and 28 min and at ambient condition of 28 ± 2°C and 85 ± 12% relative humidity and baked in an oven (Model, Convecta U/B Macadams Baking System, Cape Town, South Africa) at 220°C for 30 min. The bread samples were cooled for 1 h, then placed in low‐density polyethylene bags and kept at 24 ± 2°C.

### Determination of physical quality of bread

Weights of bread loaves were measured with a Mettler Toledo (A204) digital weighing scale. Volume was measured by millet seed displacement method (AACC 2010) with minor modification.

### Weight loss

The weight loss of the bread was determined as described by Kim et al. (2003). The dough was weighed before baking, and the breads were weighed after baking. The percent weight loss of the bread samples was calculated as:

$$\%\;\mathrm{weight}\;\mathrm{loss}=\frac{A-B\times 100}{A}$$

where:

*A = *weight of dough.

*B = *weight of baked bread.

### Sensory analysis

#### Selection of panelists

Thirty people were selected from a pool of volunteers comprising of officers, lecturers, and students of tertiary institutions. The panelists were selected after an oral interview conducted on the basis of a criteria checklist that included: good health, nonsmoker, nonallergic to wheat/banana, willingness to participate, and passion/likeness for the consumption of bread.

Quantitative acceptance test was used to assess consumers’ liking for the product. The thirty untrained panelists rated their liking or otherwise for the bread samples produced from the blends on a 9‐point hedonic scale (1 = disliked extremely as compared to reference sample ‘R’, and 9 = liked extremely as compared to reference sample ‘R’).

### Statistical analysis

All experiments and analyses were conducted in triplicates. Data obtained from different aspects of the study were subjected to analysis of variance and the Duncan multiple range test was used to separate the means (Duncan 1955). Statistical analysis package software SPSS 17 for windows was used for all the analyses.

## Results and Discussion

### Composition of the flours and their blends

Moisture content determination is an important index of quality for flour generally, and WF in particular. Moisture content above 14% affects the storage quality of flour and at more higher content, it may increase the microbial content and may also predispose the flour to infestation by insects. It may also cause the flour to agglomerate, become wet, and create production problem. The moisture content of the flour (Table 1) ranged from 9.27 ± 0.02% to 13.50 ± 0.11% and is thus, below the 14% safety limit.

**Table 1:** Effects of pretreatments on the composition of wheat‐banana composite flour

Flour	Moisture%	Protein %	Fiber %	Fat %	Ash %	CHO %	Gluten (Wet)	Alpha‐amylase min	Water absorption %
WF:100	13.50 ± 0.11_h_	11.00 ± 0.14_c_	**1.77 ± **0.01_k_	**1.38 ± **0.02_def_	**0.63 ± **0.01_ab_	71.73 ± 0.02_b_	35.64 ± 0.06_g_	4.0.00 ± 0.05_a_	51.80 ± 0.14_f_
WF:PTBF/00:100	9.92 ± 0.03_b_	5.46 ± 0.00_b_	1.54 ± 0.06_j_	2.05 ± 0.07_i_	3.87 ± 00	77.03 ± 0.04_i_	NA	39.61 ± 1.18_k_	na
WF:CTBF/00:100	9.27 ± 0.02_a_	4.75 ± 0.07_a_	0.34 ± 0.01_c_	1.27 ± 0.01_c_	2.69 ± 0.01_i_	81.62 ± 0.02_k_	NA	27.78 ± 0.11_i_	na
WF:BBF/00:100	10.32 ± 0.03_b_	4.64 ± 0.06_a_	1.36 ± 0.02_i_	1.71 ± 0.01_h_	3.00 ± 0.00_j_	78.75 ± 0.21_j_	NA	37.95 ± 0.14_k_	na
WF:PTBF/95: 05	11.80 ± 0.00_efg_	13.75 ± 0.06_k_	0.81 ± 0.01_f_	1.41 ± 0.01_efg_	0.88 ± 0.00_g_	71.35 ± 0.06_a_	35.12 ± 0.14 _h_	5.47 ± 0.21_bcd_	53.85 ± 0.21_h_
WF:CTBF/95:05	11.10 ± 0.14_c_	12.29 ± 0.07_i_	0.58 ± 0.01_d_	1.46 ± 0.01_f_	0.69 ± 0.01_bc_	73.79 ± 0.07_e_	35.09 ± 0.13_f_	5.66 ± 0.13_def_	52.70 ± 0.70_fg_
WF:BBF/95:05	11.55 ± 0.35_cdef_	12.74 ± 0.06_j_	1.06 ± 0.03 _h_	1.36 ± 0.03_cdef_	0.74 ± 0.01_cde_	72.21 ± 0.01_c_	34.07 ± 0.08_de_	5.19 ± 0.13_b_	50.75 ± 0.07_e_
WF:PTBF/90:10	11.65 ± 0.21_defg_	12.14 ± 0.07_hi_	0.67 ± 0.03_e_	1.38 ± 0.01_cdef_	0.84 ± 0.00_efg_	73.21 ± 0.29_d_	34.19 ± 0.01_de_	5.99 ± 0.13_fg_	53.55 ± 0.63_gh_
WF:CTBF/90:10	12.05 ± 0.07_fg_	11.82 ± 0.07_efg_	0.79 ± 0.01_f_	1.31 ± 0.01_cde_	0.82 ± 0.01_efg_	73.18 ± 0.07_d_	32.65 ± 0.49_cd_	5.73 ± 0.19_defg_	50.25 ± 0.071_de_
WF:BBF/90:10	11.35 ± 0.21_cdef_	12.06 ± 0.04_ghi_	0.10 ± 00_b_	1.28 ± 0.01_bcd_	0.83 ± 0.01_fg_	74.32 ± 0.14_f_	34.16 ± 21_e_	5.28 ± 0.25_b_	47.60 ± 0.14_c_
WF:PTBF/85:15	11.30 ± 0.42_cd_	11.84 ± 0.05_fg_	0.03 ± 0.01_a_	1.26 ± 0.01_b_	0.72 ± 0.00_bcd_	74.42 ± 0.28_f_	33.00 ± 0.28_d_	6.06 ± 0.11_cdg_	52.75 ± 0.07_fg_
WF:CTBF/85:15	11.10 ± 0.00_c_	11.36 ± 0.07_d_	0.83 ± 0.01_f_	1.32 ± 0.01_bcde_	0.60 ± 0.001_a_	74.92 ± 0.07_g_	32.46 ± 0.05_c_	5.87 ± 0.31_efg_	49.75 ± 0.21_d_
WF:BBF/85:15	11.50 ± 0.28_cdef_	11.92 ± 0.03_fgh_	0.92 ± 0.01_g_	1.30 ± 0.14_cde_	0.80 ± 0.14d_efg_	73.08 ± 0.07_d_	32.17 ± 0.06_c_	5.56 ± 0.11_e_	45.40 ± 0.56_b_
WF:PTBF/80:20	11.40 ± 0.00_cdef_	11.72 ± 0.40_efg_	0.32 ± 0.02_c_	1.19 ± 0.01_b_	1.22 ± 0.00_h_	74.20 ± 0.28_f_	30.25 ± 0.05_a_	6.52 ± 0.05_h_	52.60 ± 0.85_fg_
WF:CTBF/80:20	11.10 ± 0.14_c_	11.02 ± 0.03_c_	0.83 ± 0.02_f_	1.55 ± 0.07_g_	0.75 ± 0.07_cdef_	75.38 ± 0.00_h_	31.18 ± 0.43_b_	5.13 ± 0.08_ab_	48.40 ± 0.28_c_
WF:BBF/80:20	11.25 ± 0.35_cd_	11.61 ± 0.14_de_	0.05 ± 0.007_a_	1.08 ± 0.01_a_	0.72 ± 0.01_bcd_	74.88 ± 0.07_g_	30.34 ± 0.33_a_	5.41 ± 0.15_bcd_	43.10 ± 0.14_a_

‐d‐a. Mean in same column with the same subscripts are not significantly different (*P* < 0.05). WF, wheat flour; BF, banana flour from potassium (PT)‐, citric acid (CT)‐treated and blanched banana (BBF); (05–100), composite blends; n a, not applicable; NA, not available.

The treated banana flour (PTBF, CTBF, and BBF) had relatively lower protein content (4.64 ± 0.06–5.46 ± 0.00%) than the WF (11.00 ± 0.14). The pretreatment appeared to have significantly (*P* < 0.05) affected the protein content (Table 1) of the flour as the PTBF had significantly higher value than the CTBF and BBF. The protein content of treated banana flour observed in this study are more than the values reported by Zuwariah and Aziah (2009) for heat‐modified and nonmodified banana flour.

The protein contents of the flour blends ranged from 11.00 ± 0.14 to 13.75 ± 0.06 with the WF having the lowest and PTBF the highest. In all cases, protein content decreased as the banana flour was used to replace the WF in the blends. The 5% WF:PTBF had significantly higher value than the WF:CTBF/95 and WF:BBF/95, respectively, indicating that the effect of the pretreatment was more noticeable at this blending ratio. The PTBF blends had relatively higher protein values than the others in all the blending ratios except at 15% level. Aside from its nutritive values, protein content is an important criterion for selecting WF for use in bakery products. Generally, for WF, higher the protein content, the better the bread‐making characteristics of the flour. WF having high protein content is also likely to absorb more water during mixing, may also have longer mixing time, and their dough are more tolerant to over‐mixing (Bakare et al. 2015). However, this may not necessarily be applicable to composite flour with high protein content as shown in Table 1.

The fiber content of the treated banana flour ranged from 0.34 ± 0.01% to 1.54 ± 0.06% and was significantly lower than that of the WF. Crude fiber decreased as the WF was replaced by the treated flour for the various pretreatments with the PTBF and CTBF having significantly higher values at 5% and 10–20% substitution level, respectively.

The fat content of the WF was significantly (*P* > 0.05) lower than that of the treated flour, except for CTBF. The fat content of the PTBF containing blends was higher in the 5% and 10% blend, but lower at subsequent substitution levels except at 20%. In all, the fat contents of the blends ranged from 1.08 ± 0.01 to 1.46 ± 0.01 with the WF:CTBF/95:05 and WF:BBF/80:20 having the highest and lowest values, respectively.

Flour from the treated banana had significantly higher ash contents than the WF and this resulted in significant (*P* < 0.05) increase in the ash content of the blends at all levels of substitution except that of WF:CTBF/85:15.

The carbohydrate content of the pretreated flour ranged from 77.03 ± 0.04% to 81.62 ± 0.02% and was significantly (*P* < 0.05) lower than the value reported by Zuwariah and Aziah (2009), with the CTBF and PTBF the highest and lowest values, respectively. The carbohydrate content of the blends increased as WF was substituted for the treated banana flour.

The gluten content of the blends ranged from 30.25 ± 0.05% to 35.64 ± 0.06% with the WF and the PTBF/80:20 having the highest and lowest values, respectively. It also decreased in all the blends as WF was replaced with flour from the treated banana. This is to be expected since banana does not have any gluten Braganza and Tolentino 2011) and agreed with reports from previous studies (Biljan and Bojana 2008; Manuel et al. 2008; Alex et al. 2008; Ade‐Omowaye et al. 2008; Lin et al. 2009; Adeleke and Odedeji 2010; Malomo et al. 2011, 2013).

Many enzymes are naturally present in flour but only few of them have active influence on bakary operation. Alpha amylase (*α*‐amylase) is one such few enzymes. *α*‐amylases are endoenzymes that randomly hydrolyze the a‐(1,4)‐linkages in the starch polymers, generating low molecular weight dextrin (Hoseney 1994; Bowles 1996). Although, maltogenic and other maltooligosaccharide‐producing *α*‐amylases also act on the a‐(l,4)‐bonds of starch, alpha‐ amylase activity in flour is measured in Falling number values. Flour from the pretreated banana had significantly (*P* < 0.05) higher Falling number values (27.78 ± 0.11–39.61 ± 1.18) than the WF (4.0.00 ± 0.05). However, the effect of this was not pronounced in the composite blends suggesting that the flour either possesses little or no endogenous alpha‐amylase, the long outer amylopectin chains in banana starch (Zuwariah and Aziah 2009) may have resisted the liquefying effect of the alpha‐amylase, or that the pretreatment may have either inactivated or limited the activity of the enzyme. Alpha‐amylase activity in the blends ranged from 4.0.00 ± 0.05 to 6.52 ± 0.05 with the WF and the WF:PTBF/80:20 having the lowest and highest values, respectively. This implied that the extent of liquefaction and diastatic activity of the starches in the blends decreased as the proportion of the flour from the treated banana was increased (Schiller 1984; Watson 1984; Bakare et al. 2015).

Water absorption of the composite flour blends ranged from 43.10 ± 0.14 to 53.85 ± 0.21 with the WF:PTBF/95: 05 and WF:BBF/80:20 having the highest and lowest values, respectively. Also, water absorption decreased as WF was replaced with flour from each of the treated bananas which was at variance with reports of earlier studies on sweet‐potato flour (Malomo et al. 2011) and breadfruit flour (Olatunji and Akinrele 1978; Bakare et al. 2015).

### Rheological characteristics

Rheology is the science of the deformation and flow of matter. It is the study of the manner in which materials respond to applied stress or strain (Mirsaeedghazi et al. 2008). The rheological properties of food materials measured or tested by rheometers like rapid visco analyser, and alveograph provides empirical information that correlates well with actual quality results for bakery products.

### Viscosity characteristics

Banana is a high moisture starchy food, but also contains polyphenolic compounds that change the color of the fruit few hours after peeling. The pretreatments were meant to prevent enzymic browning that may negatively affect product quality. The pretreatments may also modify the properties of the banana starch in different ways.

Pasting temperature gives an indication of temperature required to cook the flour beyond its gelatinization point (BeMiller 2011). It corresponds to the temperature where viscosities first increase by at least 2 RVU over a 20 sec period (Olkku and Rha 1978; Appelqvist and Debet 1997). Changes that may occur when a starch–water system is heated, included enormous swelling, increased viscosity, translucency and solubility, and loss of anisotropy (birefringence) (Shimelis et al. 2006). Pasting temperature of the flour from the treated banana ranged from 84.70 ± 0.28 to 86.45 ± 0.00 with the WF:BBF/00:100 and the WF:PTBF/00:100 having the highest and lowest values, respectively. Flour from the chemically pretreated flour were significantly different from the blanched samples and both cooked at significantly lower temperature than the WF.

Peak viscosity is the maximum viscosity developed during or soon after the heating phase of the test. It occurred after most of the granule swelling had ceased. Hot starch paste is a mixture of swollen starch granules and granule fragments, together with colloidal and molecularly dispersed starch molecules. It gelatinizes when heated beyond 50°C. This caused a marked increase in the viscosity and further disintegration of the starch granules. The viscosity of the starch paste dropped at elevated temperature near 95°C depicting the characteristic peak in the viscosity‐temperature curve of the RVA graph (Dengate 1978; Bakare et al. 2012). The peak viscosity also measures the alpha‐amylase activity and other contributory factors such as the inherent susceptibility of the starch to amylase and the starch gel strength (Watson 1984; Meera 2010). Therefore, a higher value of RVU at the peak of the curve indicated a lower diastatic activity and vice versa (Schiller 1984). The peak viscosity of WF (186.17 ± 0.71 RVU) was found to be significantly lower than that of the treated flour (344.25 ± 12.74–461.0 ± 5.07) (Table 2). This showed that the WF had a relatively higher diastatic activity and lower gel strength than the flour from the treated banana. It also suggested that the gel strength of the composite flour and not the alpha‐amylase activity was what was measured by the Hagberg falling numbers instrument (Table 1). The peak viscosity of the flour from the pretreated banana was also significantly different from each other. Peak viscosities occur at equilibrium between swelling of the granules (that increases the viscosity) and the granule rupture and alignment (that reduces viscosity). The relatively high swelling capacity exhibited by the treated flour may have resulted from a weak internal bonding in the starch granules.

**Table 2:** Effects of pretreatments on the pasting characteristics of wheat and pretreated banana flour

Flours	Peak viscosity (aRVU)	Holding strength (RVU)	Breakdown viscosity (RVU)	Final viscosity (RVU)	Setback viscosity (RVU)	Peak time (min)	Pasting temp (°C)
WF: 100	186.17 ± 0.71^a^	128.25 ± 1.18^a^	67.92 ± 0.47^a^	217.08 ± 1.41^a^	88.83 ± 0.24^c^	6.24 ± 0.05^c^	87.20 ± 0.00^c^
WF:PTBF/00:100	461.0 ± 5.07^d^	308.13 ± 4.77^c^	152.88 ± 9.84^c^	348.5 ± 8.84^d^	40.38 ± 4.07^a^	5.14 ± 0.09^ab^	84.70 ± 0.28^a^
WF:CTBF/00:100	412.42 ± 6.13^c^	248.79 ± 3.95^b^	163.63 ± 2.18^c^	287.71 ± 9.37^b^	38.92 ± 5.42^a^	5.04 ± .05^a^	84.88 ± 0.035^a^
WF:BBF/00:100	344.25 ± 12.74^b^	245.13 ± 5.60^b^	99.13 ± 7.13^b^	312.88 ± 4.89^c^	67.75 ± 0.71^b^	5.30 ± 04^b^	86.45 ± 0.00^b^

‐d‐a. Mean in same column with the same superscripts are not significantly different (*P* < 0.05). WF, wheat flour; BF, banana flour from potassium (PT)‐, citric acid (CT)‐treated and blanched banana (BBF); (05–100), composite blends.

a 1RVU is approximately 12 cP.

Holding strength indicated the ability of the starch granules to maintain their gelatinized structure when the paste was held at 95°C for 2 min 30 sec under mechanical shearing stress. The treated flour had significantly higher holding strength value than that of WF, and were also significantly different from each other.

Breakdown viscosity is a measure of the degree of starch disintegration. It is an indication of hot paste stability of the starch. The smaller the breakdown viscosity, the higher the paste stability (Hugo et al. 2000; Bakare et al. 2012). The WF had significantly lower (67.92 ± 0.47 RVU) breakdown viscosity value than flour from the banana (99.13 ± 7.13–163.63 ± 2.18 RVU) indicating that the WF and the blanched samples have relatively better hot paste stability than the chemically treated samples (Table 2).

Final viscosity is the section of the paste gel curve where the gelatinized dispersion of starch becomes viscoelastic on cooling, resulting in the formation of a loose paste or gel. The final viscosity of flour from the treated banana ranged from 287.71 ± 9.37 to 348.5 ± 8.84 RVU; they are significantly different from each other and have significantly higher values than the WF. This indicated that the banana flour formed a firmer gel after cooking and cooling.

Setback viscosity is the phase of the pasting curve after cooling the starches to 50°C. This stage involved reassociation, retrogradation, or reordering of starch molecules. Also, the water previously bounded in the viscoelastic gel are released at this stage in a process referred to as syneresis. Higher the setback viscosity, greater is the tendency toward retrogradation. The blanched sample (WF:BBF/00:100) had more tendency toward retrogradation than the chemically treated sample, but both were better than the WF in this regard.

Peak time was the time at which the peak viscosity was attained in minutes. The WF had a significantly higher peak time than flour from the treated banana.

### Alveograph

**Figure 1:**
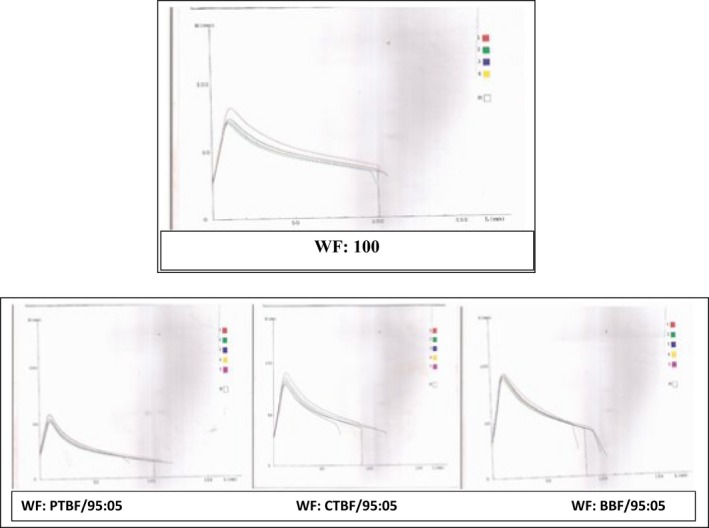
Alveogram characteristics of wheat flour and flour from composite containing 5% of pretreated banana.

The alveograph is an important dough testing instrument used to evaluate the quality of WFs for bread and biscuit and cookie making (Bettge et al. 1989; Janssen et al. 1996). It measures the resistance to expansion and the extensibility of a dough by providing the measurement for maximum over pressure, average abscissa at rupture, index of swelling and deformation energy (Fig. 1) of dough (Indrani and Rao 2007). It impacts strain rates of 0.1–1 per second, which are about 100‐fold higher than those occurring in actual baking processes (Chin and Campbell 2005).

**Figure 2:**
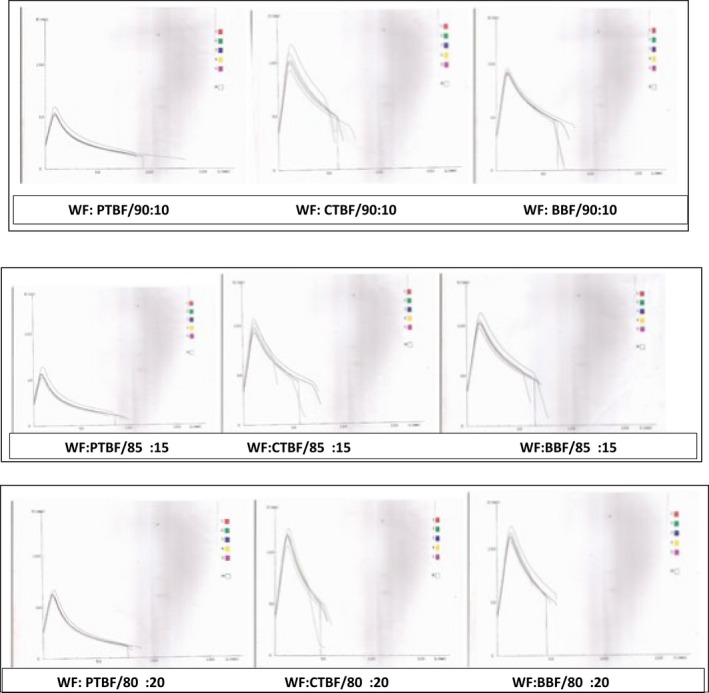
Alveogram characteristics of wheat flour and flour from composites containing 10, 15, and 20% of pretreated banana.

The peak height (*P*) indicated the resistance that the dough offered to deformation and it is related to the tensile strength or stability that the dough exhibited during the proofing stage of bread making (Pyler 1988; Mepba et al. 2007). The *P* values ranged from 61.50 ± 0.71 to 128.50 ± 0.71 mm with the WF and the WF: CTBF/80:20 blend offering the least and highest resistance to expansion, respectively (Table 3). Resistance to expansion increased in the respective blends as the WF was replaced with flour from the treated banana, but samples containing the blanched samples (Figs. 1, 2) have significantly higher *P* values at each of the levels of substitution. The *P* values of the WF was, however, significantly different from that of the blends.

**Table 3:** Effects of pretreatments on the alveogram characteristics of wheat‐banana composite flour

Flours	Peak height (mm) (*P*)	Length (mm) (*L*)	Energy (10^−4^J) (*W*)	Curve configuration (*P*/*L*)	Maximum inflation (*G*)	Elasticity index (%) (*Ie*)
WF:100	82.50 ± 0.71_d_	100.50 ± 0.71_h_	325.00 ± 1.41 _j_	0.83 ± 0.01_a_	22.35 ± 0.07_gh_	70.50 ± 0.71_l_
WF:PTBF/95: 05	61.50 ± 0.71_a_	103.00 ± 1.41_i_	186.50 ± 0.71_d_	0.65 ± 0.07_a_	22.55 ± 0.07_h_	51.40 ± 0.14_e_
WF:CTBF/95:05	91.50 ± 0.71_e_	91.50 ± 0.70_f_	321.50 ± 0.71_i_	1.00 ± 0.00_ab_	21.30 ± 0.14_ef_	67.75 ± 0.07_j_
WF:BBF/95:05	100.50 ± 0.71_f_	85.50 ± 0.71_e_	337.50 ± 0.71_k_	0.59 ± 0.83_a_	21.00 ± 0.70_e_	68.70 ± 0.14_k_
WF:PTBF/90:10	61.00 ± 1.41_a_	95.00 ± 1.41_g_	157.00 ± 1.41_c_	0.65 ± 0.01_a_	21.75 ± 0.21_fg_	45.35 ± 0.35_c_
WF:CTBF/90:10	113.50 ± 0.70_i_	57.50 ± 0.71_b_	274.50 ± 0.71_g_	1.96 ± 0.01_cd_	17.50 ± 0.70_c_	62.50 ± 0.28_h_
WF:BBF/90:10	103.25 ± 0.35_g_	65.50 ± 0.71_c_	273.50 ± 0.71_g_	0.79 ± 1.1_a_	17.85 ± 0.07_c_	64.80 ± 0.14_i_
WF:PTBF/85:15	63.50 ± 0.70_b_	86.50 ± 0.71_e_	145.50 ± 0.71_b_	0.74 ± 0.01_a_	20.70 ± 0.14_e_	40.30 ± 0.14_b_
WF:CTBF/85:15	108.50 ± 0.71_h_	56.50 ± 0.71_b_	240.50 ± 0.71_f_	1.93 ± 0.01_cd_	16.80 ± 0.14_b_	57.35 ± 0.07_g_
WF:BBF/85:15	114.00 ± 0.00_i_	64.00 ± 0.00_c_	293.00 ± 0.00_h_	1.78 ± 0.00_bc_	17.80 ± 0.00_c_	62.10 ± 0.00_h_
WF:PTBF/80:20	69.50 ± 0.71c	77.50 ± 0.70_d_	141.50 ± 0.71_a_	0.90 ± 0.00_a_	19.55 ± 0.07_d_	35.85 ± 0.07_a_
WF:CTBF/80:20	128.50 ± 0.71_k_	47.50 ± 0.71_a_	240.50 ± 0.71_f_	2.73 ± 0.01_d_	15.35 ± 0.07_a_	50.65 ± 0.07_d_
WF:BBF/80:20	124.50 ± 0.71_j_	46.50 ± 0.71_a_	237.50 ± 0.71_e_	2.75 ± 0.07_d_	15.40 ± 0.42_a_	55.75 ± 0.21_f_

‐d‐a. Mean in same column with the same superscripts are not significantly different (*P* < 0.05). WF, wheat flour; BF, banana flour from potassium (PT)‐, citric acid (CT)‐ treated and blanched banana (BBF); (05–100), composite blends.

The length (*L*) indicated the extensibility of the dough. The *L* values ranged from 46.50 ± 0.71 to 103.00 ± 1.41 mm with the WF:PTBF/95:05 and WF:BBF/80:20 having the highest and least extensibility, respectively. The *L* values decreased significantly with substitution levels with the WF:BBF being the least extensible at 5% level while at subsequent levels the WF:CTBF was the least extensible.

The *P*/*L* (configuration ratio) ranged from 0.59 ± 0.83 to 2.75 ± 0.07 with the WF:BBF/95:05 and WF:BBF/80:20 having the lowest and highest values, respectively. The *P*/*L* values increased as the WF was replaced with flour from each of the treated banana, respectively. There were no significant (*P* < 0.05) differences between the WF and the WF:PTBF (5–20%), WF:CTBF (5%), and WF:BBF (5–10%). The curve configuration ratio (*P*/*L*) is an index of gluten behavior. However, the strength of composite flour are influenced by considerations other than gluten behavior. Obviously, the significantly higher *P*/*L* values of WF:CTBF/90:10, WF:CTBF/85:15, WF:CTBF/80:20, WF:BBF/85:15, and WF:BBF/80:20 relative to other blends and the WF could not have been due to the effect of gluten as shown in Table 1. These implied that other factors that influenced the rheological attributes of the dough were probably at play and these factors may not have been clearly/succinctly captured by the test method or the interpretation of the values.

The area under the curve represents the energy required to expand the dough and is related to the baking strength of the flour. This area is generally much larger for hard WFs than for soft WFs. It ranged from 141.50 × 10^−4^J to 337.50 × 10^−4^J WF:BBF/95:05 and WF:PTBF/80:20 having the highest and lowest values, respectively. Except for WF:BBF/95:05, baking strength decreased as WF was replaced with flour from the treated banana. The energy required to expand the dough for each of the three categories of blends decreased significantly with levels of substitution.

The *G* values (Table 3) depicted the relative abilities of the dough to be inflated for maximum development until they eventually burst. It is a measure of the magnitude of the total response of the dough to the biaxial stress and strain imposed on it by the instrument. It ranged from 15.35 ± 0.07 to 22.55 ± 0.07 with the WF:CTBF/80:20 and WF:PTBF/95: 05 having the lowest and highest values, respectively. It also decreased significantly as the WF was replaced with the treated flour. The WF:PTBF/95:05 and the WF had higher values than other blends but there was no significant difference between the WF and WF:PTBF (5–10%).

Elasticity index (*Ie*) may be used to characterize the dough on the basis of the elastic resistance that they offer during their biaxial deformation (Pyler 1988; Banu et al. 2011).

It also decreased with substitution levels and the WF offered significantly better elastic resistance relative to other blends. The WF:BBF had significantly higher elasticity index at each levels of substitution than the other blends that contained flour from each of the treated bananas.

**Figure 3:**
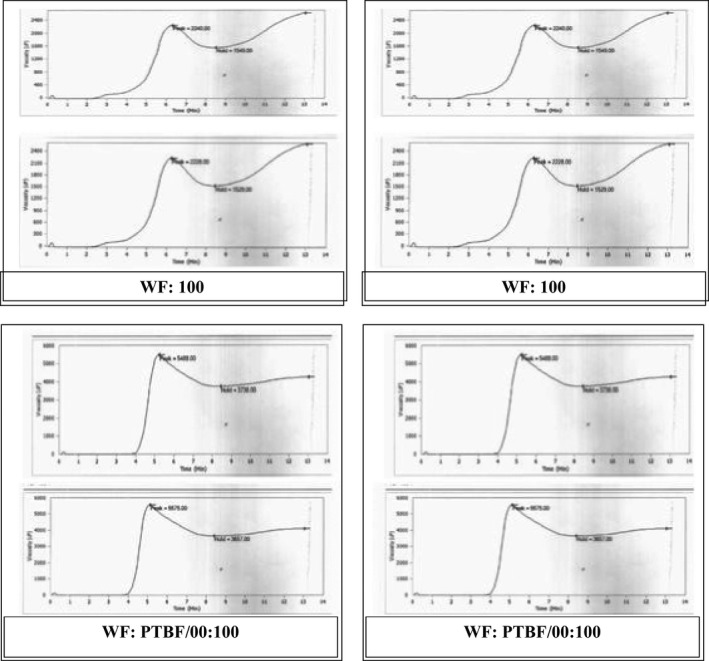
Pasting profile of wheat flour and flour from pretreated banana.

The nonlinear viscoelastic behavior of WF dough has been attributed to the continuous gluten matrix and starch granules embedded in it (Collar et al. 2007). It possesses the properties of both solid and liquid bodies, and exhibited the rheological properties that were in between that of the ideal solid and fluid bodies. The inclusion of banana flour in the blends was expected to increase the carbohydrate starch content while decreasing the quantity and quality of protein needed to sustain the viscoelastic behavior of the dough. Each of the pretreatments given to the bananas affected their composition (Table 1), pasting characteristics (Fig. 3), and the rheological characteristics of the dough made from their blends with the WF as captured by the alveograph (Figs. 1, 2).

### Evaluation of bread quality

#### Absorbed water by dough

The quantity of water used to form the dough for baking (Table 4) ranged from 48.12 ± 0.07 to 52.60 ± 0.14% with the WF:CTBF/80:20 and WF having the lowest and highest values, respectively. The WF was significantly (*P* < 0.05) different from the blends. Actual water absorption decreased as the WF was replaced by each of the flour from the treated bananas and there was no significant difference between the blends at each of the substitution ratios.

**Table 4:** Effects of pretreatments on the physical quality characteristics of wheat‐banana composite flour

Flours	Actual water absorption (%)	Dough weight (g)	Loaf height	Loaf weight (g)	Weight loss (%)	Loaf volume (cm^3^)	Loaf‐specific volume (cm^3^/g)
WF:100	52.60 ± 0.14^e^	327.0 ± 0.00^a^	10.75 ± 0.35^f^	271.65 ± 0.21^a^	16.93 ± 0.06^f^	1531.70 ± 49.24^f^	5.635 ± 0.18^f^
WF:PTBF/95: 05	51.52 ± 0.05^d^	327.0 ± 0.00^a^	11.20 ± 0.14^g^	281.05 ± 1.34^g^	14.45 ± 0.21^a^	1636.02 ± 7.07^g^	5.865 ± 0.01^g^
WF:CTBF/95:05	51.47 ± 0.03^d^	327.0 ± 0.00^a^	8.50 ± 0.14^bc^	280.10 ± 0.70^f^	14.47 ± 0.04^a^	1144.95 ± 0.04^c^	4.085 ± 0.01^bc^
WF:BBF/95:05	51.51 ± 0.08^d^	327.5 ± 0.00^b^	9.85 ± 0.07^e^	271.90 ± 0.70^ab^	17.50 ± 0.71^g^	1411.49 ± 0.21^e^	5.250 ± 0.07^e^
WF:PTBF/90:10	50.38 ± 0.01^c^	327.0 ± 0.00^a^	9.45 ± 0.07^d^	278.00 ± 0.70^e^	15.24 ± 0.14^bc^	1394.85 ± 7.1^e^	5.090 ± 0.07^e^
WF:CTBF/90:10	50.33 ± 0.02^c^	327.0 ± 0.00^a^	8.25 ± 0.07^b^	277.85 ± 0.07^e^	15.06 ± 0.01^b^	1157.68 ± 0.71^c^	4.175 ± 0.01^bc^
WF:BBF/90:10	50.34 ± 0.04^c^	327.0 ± 0.00^a^	6.35 ± 0.07^a^	276.70 ± 0.14^cde^	15.43 ± 0.03^bcd^	774.77 ± 0.01^a^	2.850 ± 0.07^a^
WF:PTBF/85:15	49.34 ± 0.14^b^	327.0 ± 0.00^a^	8.45 ± 0.21^bc^	278.50 ± 1.41^e^	15.24 ± 0.14^bc^	1223.82 ± 0.14^d^	4.415 ± 0.01^d^
WF:CTBF/85:15	49.28 ± 0.09^b^	327.0 ± 0.71^a^	8.26 ± 0.01^b^	275.75 ± 0.071^cd^	15.70 ± 0.01^cd^	1164.25 ± 0.01^cd^	4.225 ± 0.01^c^
WF:BBF/85:15	49.27 ± 0.07^b^	327.0 ± 0.00^a^	8.65 ± 0.07^c^	273.50 ± 0.14^b^	16.39 ± 0.01^e^	1222.07 ± 0.01^d^	4.475 ± 0.01^d^
WF:PTBF/80:20	48.18 ± 0.09^a^	327.0 ± 0.00^a^	8.35 ± 0.21^bc^	277.40 ± 1.55^de^	15.46 ± 0.06^bcd^	1226.55 ± 24.57^d^	4.495 ± 0.16^d^
WF:CTBF/80:20	48.12 ± 0.07^a^	327.0 ± 0.00^a^	8.15 ± 0.07^b^	277.25 ± 0.071^cde^	15.24 ± 0.01^bc^	1131.43 ± 0.03^bc^	4.185 ± 0.01^bc^
WF:BBF/80:20	48.14 ± 0.06^a^	327.0 ± 0.00^a^	8.26 ± 0.01^b^	275.55 ± 0.21^c^	15.79 ± 0.01^d^	1104.85 ± 0.01^b^	4.020 ± 0.01^b^

‐d‐a. Mean in same column with the same superscripts are not significantly different (*P* < 0.05). WF, wheat flour; BF, banana flour from potassium (PT)‐, citric acid (CT)‐treated and blanched banana (BBF); (05–100), composite blends.

#### Specific volume

Loaf volume is used as a criterion to measure the quality of fresh bread in research quality control in industry and by consumers (Penfield and Campbell 1990; Zuwariah and Aziah 2009). Specific volume of loaves of bread provide a uniform basis for comparing results of various studies (Oyeku et al. 2008; Bakare et al. 2015). It ranged from 2.850 ± 0.07 to 5.865 ± 0.01 cm^3^/g with the WF:PTBF/95: 05 having significantly higher specific volume than other blends. The values decreased as flour from the treated banana replaces the WF in the blends but the 5% WF:PTBF was significantly (*P* > 0.05) better than the WF.

In WF, specific volume is an indication of the gluten content of the bread (Van Hall 2000; Abang Zaidel et al. 2010) but other constituents such as starch and fiber also contribute to the specific volume of bread. Gluten or more precisely glutenin, is the main structure‐forming protein in WF that is responsible for the elastic and extensible properties needed to produce good quality wheat bread (Bloksma 1990b; Gallagher et al. 2003). Bread made from soft WF usually yield lower loaf volumes. It has also been shown that the difference between weak and strong flours can be explained by differences in the molecular mass distribution of their proteins (MacRitchie 1973). Abundance of glutenin molecules with long chain was observed to have made the protein phase, and consequently the dough, highly extensible (Bloksma 1990a). Differences in the pasting temperature and peak viscosity of composite starches have also been suspected to influence extensibility (Greene and Bovell‐Benjamin 2004).

#### Weight loss

Cut‐out dough losses weight during the proofing and baking stages of bread processing. This may be due to both fermentation losses brought about by amylases of starch and utilization of soluble sugar by yeast, and also by evaporation of moisture during baking. Weight loss ranged from 14.45 ± 0.21 to 17.50 ± 0.71%. The WF:PTBF/95:05 lost the most weight while the WF:BBF/95:05 lost the least weight. Except for the blends that contain the blanched sample, weight loss increased as the flour from the treated bananas replaces the WF in the blends (Table 4).

#### Sensory evaluation

Sensory scores ranged from 3.87 ± 1.81, 5.16 ± 1.42, 4.77 ± 1.76, 5.13 ± 1.69, and 4.70 ± 1.68 to 8.27 ± 0.64, 7.83 ± 1.05, 7.30 ± 1.24, 8.03 ± 1.03, and 7.87 ± 0.93 for appearance, flavor, aroma, texture, and taste, respectively (Table 5). Bread samples prepared from the WF had significantly higher scores for all the sensory attributes while the WF:BPT/80:20 scored the lowest in appearance, aroma, texture, and the WF:CTPT/80:20, the lowest score for flavor and taste, respectively.

**Table 5:** Effects of pretreatments on the sensory quality characteristics of wheat‐banana composite flour

Flours	Appearance	Flavor	Aroma	Texture	Taste
WF: 100	8.27 ± 0.64 ^g^	7.83 ± 1.05^c^	7.30 ± 1.24^d^	8.03 ± 1.03^c^	7.87 ± 0.93^e^
WF:PTBF/95: 05	6.30 ± 1.56^ef^	5.83 ± 1.63^ab^	5.53 ± 1.81^abc^	6.70 ± 1.51^b^	6.33 ± 1.35^cd^
WF:CTPT/95:05	6.63 ± 1.03^f^	6.26 ± 1.57^b^	5.77 ± 1.65^bc^	6.40 ± 1.33^b^	6.53 ± 1.46^d^
WF:BPT/95:05	5.83 ± 1.76^def^	5.87 ± 1.43^ab^	5.70 ± 1.21^bc^	5.83 ± 1.80^ab^	6.03 ± 1.40^cd^
WF:PTBF/90:10	5.63 ± 1.65^cde^	5.90 ± 1.45^ab^	5.63 ± 1.69^abc^	5.40 ± 2.13^a^	6.13 ± 1.68^cd^
WF:CTPT/90:10	5.53 ± 1.33^cde^	5.67 ± 1.83^ab^	5.97 ± 1.27^c^	5.77 ± 1.79^ab^	6.50 ± 1.38^d^
WF:BPT/90:10	5.47 ± 1.96^bcde^	6.00 ± 1.11^ab^	5.77 ± 1.43^bc^	6.10 ± 2.01^ab^	6.13 ± 1.61^cd^
WF:PTBF/85:15	4.67 ± 1.92^abc^	5.57 ± 1.38^ab^	5.33 ± 1.56^abc^	5.27 ± 1.66^a^	5.47 ± 1.63^abc^
WF:CTPT/85:15	5.17 ± 1.72^bcd^	5.90 ± 1.56^ab^	5.57 ± 1.55^abc^	5.93 ± 1.51^ab^	5.63 ± 1.61^bcd^
WF:BPT/85:15	4.50 ± 2.21^ab^	5.33 ± 1.71^ab^	5.03 ± 1.83^ab^	5.87 ± 1.89^ab^	5.47 ± 1.66^abc^
WF:PTBF/80:20	4.80 ± 1.94^abc^	5.33 ± 1.75^ab^	4.93 ± 1.57^ab^	5.13 ± 1.69^a^	5.03 ± 1.92^ab^
WF:CTPT/80:20	4.50 ± 1.66^ab^	5.16 ± 1.42^a^	4.93 ± 1.53^ab^	5.17 ± 1.68^a^	4.70 ± 1.68^a^
WF:BPT/80:20	3.87 ± 1.81^a^	5.33 ± 1.92^ab^	4.77 ± 1.76^a^	5.33 ± 1.88^a^	5.47 ± 2.17^abc^

‐d‐a. Mean in same column with the same superscripts are not significantly different (*P* < 0.05). WF, wheat flour; BF, banana flour from potassium (PT)‐, citric acid (CT)‐treated and blanched banana (BBF); (05–100), composite blends.

In the blends containing flour from the treated samples, the WF:PTBF/95:05 had better scores for appearance, texture, and taste while the WF:CTPT/95:05 had better scores for flavor and aroma, respectively.

## Conclusion

The main aim of this study was to produce bread from composite flour comprising of banana and wheat flour without having to alter considerably, the rheological attributes of the dough and the quality attributes associated with bread from wheat flour. These necessarily involve the conversion of the banana to flour while controlling the undesirable enzyme induced discoloration associated with the use of banana flour. That was why it was necessary to evaluate how the pretreatments that were primarily meant to mitigate the discoloration effect of the enzyme activities modified the composition, rheology, and baking qualities of the flour.

The study concluded that the pretreatments have significant effects on the composition of the banana flour and also have varying effects on the composition and pasting properties of their composite flour with WF. For instance, the PTBF had significantly higher protein value than the CTBF and BBF, while the PTBF containing blends had relatively higher protein values than other blends except at 15% level. Crude fiber of the blends decreased as the WF was replaced by the treated flour for the various pretreatments with the PTBF and CTBF having significantly higher values at 5% and 10–20% substitution level, respectively.

Flour from the pretreated banana had significantly (*P* < 0.05) higher Falling number values implying that the pretreatments may have (since the study design did not include untreated banana flour) reduced the alpha‐ amylase activity of the treated flour thereby decreasing the extent of liquefaction and diastatic activity of their starches. This observation reflected in the blends as the proportion of the flour from the treated banana was increased.

Also, contrary to reports of earlier studies on sweet‐potato and breadfruit flour, water absorption decreased as WF was replaced with flour from each of the treated bananas. This indicated that the pretreatments modified the pasting properties of the banana starch in several ways. For instance, the pasting temperature of the chemically pretreated flour was significantly different from the blanched samples and both cooked at significantly lower temperature than the WF. Similarly, the peak viscosity of the flour from the pretreated banana were also significantly different from each other.

The rheological attributes of the composite flour in which they were included was also affected in different ways at various levels of inclusion in their composite with WF. For instance, the curve configuration ratio (*P*/*L*), which is an index of the rheological attributes of the dough, showed that composite flour containing the potassium‐treated flour (WF:PTBF) was not significantly different from the WF at all the levels of inclusion (5–20%) in this study, while those containing the blanched sample (WF:BBF) and the citric acid‐treated (CTBF) sample were only comparable to the WF at 5% and up to 10% levels of inclusions, respectively. The specific volume of WF:PTBF bread was significantly (*P* > 0.05) better than the bread produced from the WF and had better scores for appearance, texture, and taste than bread samples from other blends.

## Conflict of Interest

None declared.
